# Biomarkers in Nephropathic Cystinosis: Current and Future Perspectives

**DOI:** 10.3390/cells11111839

**Published:** 2022-06-04

**Authors:** Francesco Emma, Giovanni Montini, Marco Pennesi, Licia Peruzzi, Enrico Verrina, Bianca Maria Goffredo, Fabrizio Canalini, David Cassiman, Silvia Rossi, Elena Levtchenko

**Affiliations:** 1 Department of Pediatric Subspecialties, Division of Nephrology, Bambino Gesù Children’s Hospital-IRCCS, 00165 Rome, Italy; francesco.emma@opbg.net; 2 Pediatric Nephrology, Dialysis and Transplant Unit, Fondazione Ca’ Grande IRRCS Ospedale Maggiore Policlinico, 20122 Milan, Italy; giovanni.montini@unimi.it; 3 Department of Clinical Sciences and Community Health, University of Milan, 20122 Milan, Italy; 4 Institute for Maternal and Child Health, IRCCS Burlo Garofolo, 34137 Trieste, Italy; pennesi@burlo.trieste.it; 5 Pediatric Nephrology Unit, Regina Margherita Children’s Hospital, AOU Città della Salute e della Scienza di Torino, 10126 Turin, Italy; licia.peruzzi@unito.it; 6 Dialysis Unit, Department of Pediatrics, IRCCS Istituto Giannina Gaslini, 16147 Genoa, Italy; enricoverrina@ospedale-gaslini.ge.it; 7 Department of Pediatric Subspecialties, Division of Metabolic Diseases, Bambino Gesù Children’s Hospital-IRCCS, 00165 Rome, Italy; biancamaria.goffredo@opbg.net; 8 Medical Department, Chiesi Pharmaceutics, 43100 Parma, Italy; f.canalini@chiesi.com (F.C.); si.rossi@chiesi.com (S.R.); 9 Department of Metabolic Diseases, University Hospitals Leuven, 3000 Leuven, Belgium; david.cassiman@uzleuven.be; 10 Department of Pediatric Nephrology and Development and Regeneration, University Hospitals Leuven, University of Leuven, 3000 Leuven, Belgium

**Keywords:** nephropathic cystinosis, biomarkers, cystine, cysteamine, kidney, therapeutic monitoring

## Abstract

Early diagnosis and effective therapy are essential for improving the overall prognosis and quality of life of patients with nephropathic cystinosis. The severity of kidney dysfunction and the multi-organ involvement as a consequence of the increased intracellular concentration of cystine highlight the necessity of accurate monitoring of intracellular cystine to guarantee effective treatment of the disease. Cystine depletion is the only available treatment, which should begin immediately after diagnosis, and not discontinued, to significantly slow progression of renal and extra-renal organ damage. This review aims to discuss the importance of the close monitoring of intracellular cystine concentration to optimize cystine depletion therapy. In addition, the role of new biomarkers in the management of the disease, from timely diagnosis to implementing treatment during follow-up, is overviewed.

## 1. Introduction

Cystinosis is a rare lysosomal storage disorder caused by autosomal recessive mutations in the *CTNS* gene that encodes the cystine transporter cystinosin, a ubiquitously expressed lysosomal cystine–proton co-transporter, which is expressed at the lysosomal membrane and mediates the efflux of cystine from the lysosome [1,2,3]. *CTNS* gene mutations lead to a deficiency or absence of cystinosin, with consequent accumulation of free cystine in lysosomes and buildup of toxic crystals that ultimately lead to tissue and organ damage. Cystinosis is a systemic metabolic disorder that initially affects the kidneys, as well as the eyes with accumulation of corneal cystine crystals, and, subsequently, endocrine and reproductive organs, muscles, bones, lungs, skin, and the central nervous system [4].

Based on the severity of presentation and age of onset, three clinical forms of the disease can be defined: infantile or early-onset nephropathic [5], juvenile or late-onset nephropathic [6], and the adult or ocular non-nephropathic form [7,8]. At present, more than 140 mutations have been reported [9,10], with the infantile form of cystinosis being associated with severe *CTNS* mutations on both alleles, and the juvenile and ocular forms mostly being associated with milder mutations in at least one allele [8,10].

The estimated incidence of cystinosis is 1 in 100,000–200,000 live births [7,8]. Infantile nephropathic cystinosis is the most common (95% of total cases) and severe clinical form of the disease, and is associated with high morbidity and mortality. The infantile form phenotypically manifests as renal Fanconi syndrome by 6–12 months of age [8]. With time, chronic kidney disease (CKD) develops [11]. Additional clinical characteristics include poor growth and failure to thrive, severe polyuria, polydipsia and dehydration, vomiting and feeding difficulties, and vitamin D-resistant hypophosphatemic rickets in children and osteomalacia in adults. During the first months of life, patients are usually asymptomatic. However, they already demonstrate elevated amounts of amino acids, low molecular weight proteins, and glucose in their urines. These represent early, although not necessarily specific, biomarkers [12]. If untreated, nephropathic cystinosis can lead to end-stage kidney disease (ESKD) by 10–12 years of age and systemic disease with multi-organ involvement, requiring treatment with dialysis and kidney transplantation [13,14,15,16]. Delayed diagnosis may occur because of the rarity of the disease and its incomplete clinical presentation at an early age. Patients with juvenile cystinosis can mimic other proteinuric conditions or might present with impaired kidney function of unknown etiology or bone complaints, as renal Fanconi syndrome is mild compared with patients having infantile cystinosis. Nevertheless, a careful examination of those patients reveals signs of proximal tubular dysfunction (aminoaciduria, low molecular weight proteinuria, glucosuria, and phosphaturia) suggesting the possibility of cystinosis [17]. Patients with all three clinical forms (infantile, juvenile, ocular) demonstrate the pathognomonic cornea cystine crystals, allowing the immediate diagnosis of cystinosis prior to confirmation by while blood cells cystine measurements and genetic testing [5,6,7,8].

Cystine-depletion therapy with cysteamine allows significant improvement in life expectancy [14,18], but cannot prevent the development of CKD and other systemic complications, and needs to be continued after kidney transplantation [7]. Recent data have demonstrated that lysosomal cystine accumulation is not the only pathological event related to the absence of functional cystinosin [19], which might explain why treatment with cysteamine is not curative. It is, however, the only available treatment for these patients. Adequate depletion is therefore needed and since the efficacy of cystine-depletion may be different due to inter-individual variability [20], precise evaluation of patient’s cystine levels needs to be monitored in clinical practice to tailor the individual cysteamine dose adjustment. Therefore, there is an urgent need to identify novel biomarkers not only for monitoring the progression of the disease, but also its prognosis in light of understanding the role of mechanisms other than cystine accumulation in the onset of pathological events. In this review, we focus on biomarkers of cystine accumulation, which remains the key feature of cystinosis and a target of cysteamine therapy.

## 2. Methods for Measuring Intracellular Leucocyte Cystine

The quantification of intracellular cystine is important for both diagnosis and monitoring of cystinosis therapy. Different methods have been developed to measure cystine content in leucocytes, which are the best cells for these analyses because they are easily and repeatedly accessible; in these cells, the cystine concentration is increased up to 80-fold compared with normal individuals [21,22]. Historically, quantification of intracellular leucocyte cystine (ILC) was performed using cystine-binding protein derived from *E. coli* [23,24]. This method was later abandoned given its high cost and use of radioactivity. Ion exchange chromatography, HPLC, and tandem mass spectrometry (MS) [25,26,27] are currently used by different laboratories, with tandem MS being the most sensitive method.

### 2.1. Cell Isolation

One of the most critical steps in ILC determination is the isolation of cells from whole blood. Intracellular cystine mainly accumulates in phagocytic cells—polymorphonuclear (PMN) leukocytes and monocytes—and not in lymphocytes [21]. Initially, cystine was measured on mixed leukocyte samples [28]. The results, however, are less reliable because they depend on the composition of white blood cells. For example, since lymphocytes usually predominate in young children, their mixed leukocyte ILC content is usually lower and in some cases can even delay diagnosis [29].

The type of anticoagulant used in blood collection is also important, with acid citrate dextrose or heparin being preferred over EDTA [30]. Two methods have been used for PMN isolation, namely Ficoll gradient centrifugation [31], and more recently, immunomagnetic granulocyte purification [32].

After isolation, sulfhydryl exchange needs to be blocked with reagents such as N-ethylmaleimide that prevent oxidation of cysteine into cystine. After this stage, samples can be stored at −80 °C if cystine measurements will be delayed or in samples that need to be shipped. The shipping of blood samples also allows for the frequent monitoring of patients who are far from the analytical laboratory, thus improving disease control. Before measuring cystine, samples are sonicated to disrupt the lysosomal membrane and acidified with sulfosalicylic acid [26]. According to local protocols, samples that need to be shipped are pre-treated or are shipped as fresh samples and immediately processed upon arrival. In this latter case, samples should arrive at the laboratory no later than 24 h after collection.

### 2.2. Cystine and Protein Determination

The first method to quantify cystine used a cystine-binding protein assay, which allowed the detection of nanomolar concentrations of cystine by isotopic dilution [23,24]. Alternatively, ion exchange chromatography has been used. This technique, however, is less sensitive and sometimes generated false results when samples were too small [8].

Current techniques for the assessment of ILC levels include high-performance liquid chromatography (HPLC) and liquid chromatography-tandem mass spectrometry (LC-MS/MS) [25,26,27]. Both techniques require specialized personnel and specific equipment. For these reasons, they are only performed in a few specialized centers. Compared to HPLC, LC-MS/MS allows measuring ILC on smaller amounts of blood, and for this reason is increasingly used. Before measuring cystine, the protein fraction is precipitated, internal standards are added, and the final solution is extracted with acetonitrile. Centrifuged samples can be analyzed at a later time. The pellet is re-suspended to measure protein content.

ILC is expressed as nanomoles of half-cystine (each molecule of cystine is composed of 2 cysteine moieties linked by a disulfide bond) normalized per milligram of protein. Often, proteins are evaluated by the classic Lowry method [33]. The best method for determination of protein is a matter of debate. There may be some preference for the use of the bicinchoninic acid method over the Lowry method [34]. The method for protein determination is important because it affects the denominator of the ILC value and the reference range for a given laboratory. For mixed-leukocyte preparations, a correction factor of 0.65 has been proposed to compare values of ILC obtained using the bicinchoninic acid vs. the Lowry assay [35]. Each laboratory needs to establish its own reference intervals for control subjects, healthy heterozygotes, and patients at diagnosis, also considering differences in age and gender [36].

## 3. Prenatal and Neonatal Diagnosis

Diagnosis of cystinosis should always be confirmed with genetic testing. Early diagnosis is crucial as it allows the early initiation of therapy. This is important for better prognosis of long-term kidney function [37].

### 3.1. Prenatal Diagnosis

Prenatal diagnosis of cystinosis can be performed in at-risk pregnancies by molecular analysis of the *CTNS* gene in chorionic villi or circulating fetal cells. Traditionally, the quantification of cystine was performed in chorionic villi or cultured amniocytes using [^14^C]-cystine [38]. This method is no longer used and has been replaced by genetic testing, a safer, faster, and cost-effective test.

### 3.2. Neonatal Diagnosis in at Risk Siblings

When prenatal evaluation in at-risk pregnancies is not feasible, DNA testing can be performed immediately after birth. Early diagnosis before four weeks of age, followed by prompt treatment with cysteamine from the age of five weeks, can protect tubular and glomerular function, at least during the first years of life [39]. Cystine content can be measured in peripheral leucocytes in samples obtained from placenta or in fibroblasts [40]. Since cystine crystals develop more rapidly in bone morrow compared to the cornea, detection of crystals in bone marrow was used in the past for diagnosis of cystinosis [41]. While this method is rapid and can allow the identification of cystine crystals during early life before they can be detected in the cornea, the aspiration of bone marrow is highly invasive and is not recommended in routine clinical practice.

### 3.3. Newborn Screening in Unaffected Population

Cystinosis is a treatable disease. Early treatment significantly impacts long-term kidney function [37]. Several groups have investigated strategies for newborn screening. Measuring cystine concentrations in dry blood spots is not reliable due to oxidation of intracellular cysteine (authors’ unpublished observation). The quantification of the seven-carbon sugar sedoheptulose in dried blood spots has been proposed as a quick pre-symptomatic method for detection of homozygosity for the most common *CTNS* 57-kb deletion. However, this method detects patients with a mutation that is prevalent only in Northern Europe [42].

Currently, detection of *CTNS* gene pathogenic variants by next-generation sequencing (NGS) is being investigated in several laboratories. A proof-of-principle demonstration of the validity of this approach has been recently produced in Germany by combining quantitative polymerase chain reaction (qPCR) and NGS [43]. In this study, one child was diagnosed in the neonatal period and was treated immediately with cysteamine; at 16 months of age, this patient had no signs of renal Fanconi syndrome [44].

## 4. Treatment Monitoring: Importance of Target Cystine Values

**Figure 1:**
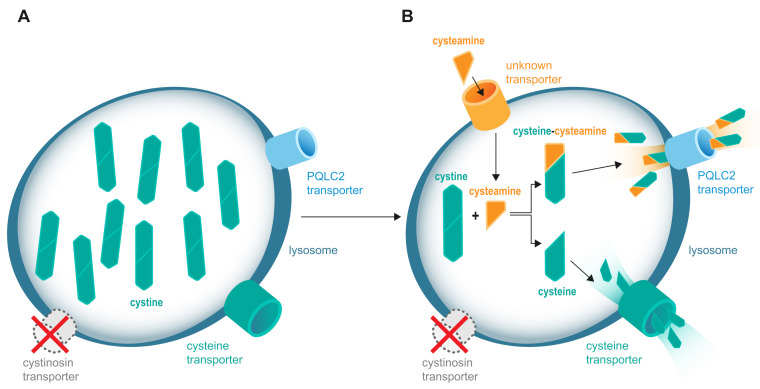
Cysteamine depletes lysosomal cystine accumulation in cystinosis. (**A**) The absence or the lack of function of the cystinosin transporter causes intracellular accumulation of cystine. (**B**) The administration of cysteamine allows the depletion of excess cystine from lysosomes in cells.

Maintenance of low ILC is currently the only way to monitor cysteamine treatment (Figure 1A,B).

Available preparations of cysteamine include immediate- and delayed-release formulations (IR-CYS and DR-CYS, respectively). The therapy is also complemented by the use of cysteamine eye drops to dissolve corneal cystine crystals, and by symptomatic treatments aiming at supplementing losses due to renal Fanconi syndrome and correcting the consequences of extra-renal intracellular cystine accumulation.

Early treatment initiation and constant monitoring of the effective cystine depletion in each patient has an impact on disease progression by protecting from cellular and tissue damage in both renal and extra-renal tissues. Oral cysteamine therapy should begin as soon as the diagnosis is made, should not be discontinued, and should be monitored for efficacy [14,45]. Early, continuous, and effective depletion therapy is able to modify the course of the disease, postponing ESKD and also reducing the incidence and severity of pathologies that are consequences of the systemic deposition of cystine, such as hypothyroidism, diabetes mellitus, neuromuscular dysfunction, and cerebral atrophy, with a general improvement in the both the quality of life and life expectancy [8,16,46,47,48,49].

Continuous cysteamine therapy shows remarkable long-term benefits as long as the treatment is adequate. The appropriateness of therapy is defined by its ability to maintain an optimal low intracellular concentration of cystine [16]. In order to delay or avoid the progression of multi-organ dysfunction and comorbidities, and to decrease mortality [50], therapeutic monitoring needs to be consistent, and dose adjustments of cysteamine should be made on the basis of ILC levels. The cystine concentration within cells of healthy individuals is below 0.2 nmol half-cystine/mg protein and below 1 nmol half-cystine/mg protein in heterozygous subjects who present the gene mutation/deletion in one allele and no clinical symptoms [51]. Homozygous patients who are not subjected to therapy have intracellular cystine levels that are >2 nmol half-cystine/mg of protein.

The optimal cut-off value for appropriate therapy is defined by most laboratories as 1 nmol half-cystine/mg of protein in mixed WBC preparations, i.e., the level seen in heterozygous individuals without clinical symptoms [52]. Cystine levels between 1 and 2 nmol half-cystine/mg of protein, although not optimal, are considered acceptable. Indeed, in some patients the optimal value cannot be reached as high cysteamine dosages are not tolerated. For concentrations > 2 nmol half-cystine/mg of protein, important clinical consequences become evident: for instance, every year of sub-optimal treatment has been shown to correspond to a loss of 0.9 years of renal glomerular function [45] (Table 1).

Of note, cystine levels in PMN leukocytes are usually higher than those in mixed leukocyte preparations, and laboratories measuring cystine in these cells might have slightly higher target values. Hence, the use of granulocyte cystine levels as a diagnostic tool or for therapeutic monitoring requires additional validation to determine what levels depict global cystine burden and proper therapeutic adherence [36]. Until consensus is reached on the use of granulocytes, it is fundamental that laboratories assessing elevated cystine levels in different leucocyte populations and with different methods provide their own reference values for diagnosis and therapeutic monitoring [47].

## 5. Limitations of Current Monitoring

While ILC is currently accepted as a gold standard for therapeutic monitoring of cystinosis, the method has several limitations related to technical and economic issues of sample measurement and storage. For instance, for reliable results, leucocytes should be isolated as soon as possible after blood collection to guarantee integrity, potentially becoming an issue for centers distantly located from the analytical laboratory. In addition, biochemical and instrumental analyses are time-consuming, and keeping analytical techniques such as LC-MS/MS routinely active may be expensive for some small centers and low-income countries, both in terms of equipment needed and personnel expertise. As previously mentioned, proper therapeutic monitoring is mandatory for follow-up given the large variability in the pharmacokinetics of cysteamine, which shows a somewhat poor correlation between the cysteamine dose and ILC levels [20,49]. Therefore, it is always necessary to quantify the actual cystine depletion to ensure that it is maintained below the validated cut-off value, and not simply rely on patient adherence to cysteamine therapy.

Moreover, because of the short lifespan of leucocytes, ILC levels may not sufficiently reflect renal and extra-renal tissue levels of cystine. Finally, with the exception of cystine in the cornea [54], there is no direct evidence that detection of cystine in other organs, such as the skin [55], can offer a reliable tool for therapeutic monitoring.

## 6. Novel Biomarkers

### 6.1. Biochemical Biomarkers

#### 6.1.1. Chitotriosidase

In order to obtain a better picture of the burden of cystine on the entire body, macrophage activation biomarkers have recently been proposed as additional therapeutic monitoring tools for cystinosis [4,56,57,58]. Macrophages are long-living cells, and their activation is an important pathogenic mechanism present in cystinosis [56,57,59]. Indeed, cystine crystals containing macrophages have been identified in several tissues, including kidney, liver, and the skin [60,61,62,63,64]. Upon activation, macrophages release high levels of inflammatory biomarkers.

A recent study established the validity of plasma chitotriosidase, an enzyme produced by activated macrophages, as a reliable biomarker for long-term therapeutic monitoring of nephropathic cystinosis [58]. In a cohort of 57 patients, this prospective international, multicenter study collected and compared the levels of ILC with four biomarkers of macrophage activation over a period of two years: interleukin (IL)-1β, IL-6, IL-18, and plasma chitotriosidase. Of these, plasma chitotriosidase levels were found to be significantly correlated with ILC levels. Specifically, a cut-off value for chitotriosidase activity of 150 nmol/ml plasma per hour was demonstrated to be both reliable and specific in distinguishing good versus poor therapeutic control compared to ILC reference values (<2 versus ≥2 nmol half-cystine/mg protein). Moreover, chitotriosidase activity was also significantly correlated with the number of extrarenal complications burdening patients. In this case, a cut-off value for chitotriosidase of 250 nmol/mL plasma per hour was found to have a specificity of 93% in identifying patients suffering from multiple extrarenal complications, thus acting as a predictor of disease severity. From a technical point of view, the use of chitotriosidase as a biomarker presents several benefits, such as the stability of chitotriosidase in plasma for longer periods of time compared to leucocytes, i.e., over 1 month at room temperature, and over 4 months when stored at +4 °C, compared to less than 24 h for leucocytes [65,66]. It is also simpler, faster, and less expensive, as well as being an easily accessible fluorometric assay compared to LC-MS/MS. The drawback of this biomarker is that about 5% of the population carry a mutation in the chitotriosidase gene, which precludes its use.

#### 6.1.2. Alpha-Ketoglutarate

Metabolomic analysis of *CTNS^−/−^* proximal tubule cell lines revealed altered metabolic pathways (glycolysis, TCA cycle, DNA replication, and DNA repair) and a reduction of lysosomal catalytic proteins expression. One of the differentially expressed metabolites is alpha-ketoglutarate (AKG), which is significantly increased in proximal tubular cells and plasma in patients with cystinosis. AKG is a key molecule in the Krebs cycle, acting as a nitrogen scavenger and a source of glutamate and glutamine that stimulates protein synthesis and inhibits protein degradation. AKG plays a pivotal role in the regulation of autophagy and apoptosis, potentially bridging the latter to the loss of cystinosin and kidney proximal tubule impairment in cystinosis. [67]. Alpha-ketoglutarate might therefore be considered a new biochemical biomarker of cystinosis.

Bicalutamide, a drug used in the treatment of prostate cancer, was able to re-establish metabolic homeostasis and reduce alpha-ketoglutarate levels in an organoid model (patient-derived tubuloids), and in cystinotic zebrafish. Although an anti-androgenic effect of bicalutamide should be taken into account, combined treatment with cysteamine might be a promising therapy that can potentially act on improving proximal tubule cell function while reducing cystine levels [67].

### 6.2. Quantification of Cystine Crystals

#### 6.2.1. Intestinal Cystine Crystals

Besides the determination in the cornea [27], cystine crystals can be detected, upon biopsy, in intestinal mucosa, where they tend to diminish following adequate cysteamine therapy. However, they are still present even when levels of cystine in white blood cells are low, thus suggesting that cysteamine treatment may not be completely effective within tissues [62]. As in other organs, cystine crystals in gastric and intestinal mucosa preferentially accumulate within interstitial macrophages [68]. Obviously, routinely performing gastric or intestinal biopsies is not suitable for therapeutic disease monitoring due to the invasiveness of the procedure and low reproducibility.

#### 6.2.2. Intradermal Cystine Crystal Determination and Skin Aging

Accelerated skin aging is characteristic of cystinosis patients [63], where cystine crystals accumulate in dermal macrophages and fibroblasts. Crystals in skin can be determined in vivo by advanced non-invasive methods, such as high-definition optical coherence tomography [69] and reflectance confocal microscopy [55]. High-definition optical coherence tomography in skin from patients with cystinosis reveals a significant reduction in epidermal and papillary dermis thickness with respect to healthy controls. Moreover, the reduced thickness of epidermis in subjects characterized by *CTNS* mutations was found to be predictive of extrarenal manifestations such as retinopathy and primary hypothyroidism [69].

Reflectance confocal microscopy allows differentiation of the density, within skin, of crystal-containing particles that are present in higher quantities in older patients and in those who started cysteamine treatment later [55]. This method, coupled with an automated and unbiased imaging tool for the quantification of crystal area and volume, makes cystine crystals in skin a novel biomarker that can facilitate long-term monitoring of cystinosis patients in clinical practice [70]. The study by Bengali et al. [70] expanded the observation of Chiaverini et al. [55], monitoring 70 cystinosis patients for over two years through analysis of punch skin biopsy and the quantification of images of 2D area and 3D volume of crystals in dermis, compared to 27 healthy controls. Images were automatically processed and showed significantly different mean values in cystinosis patients vs. controls. Moreover, the normalized confocal crystal volume increased with age and was also associated with the stage of CKD and diagnosis of hypothyroidism, thus showing its potential as a biomarker of disease severity over time [70].

Novel biomarkers for cystine are summarized in Table 2.

## 7. Conclusions

Prognosis of nephropathic cystinosis has dramatically improved due to the introduction of cystine-depleting therapy and renal replacement therapy. Patients receiving early diagnosis and early treatment have an increased life expectancy and better quality of life. Early, continuous, and appropriate cysteamine depletion therapy allows the maintenance of intracellular cystine within optimal cut-off values. Despite the limitations, leucocyte cystine measurements is the only validated tool for disease monitoring, and its use for optimization of depleting treatment is crucial. Nevertheless, it is pivotal to develop alternative strategies for therapeutic monitoring to overcome the limitations of this marker. Further research is required to validate and integrate different novel biomarkers to be used as a single tool which will allow accurate estimation of the efficiency of treatment and long-term prognosis of patients with cystinosis.

## Figures and Tables

**Table 1:** Clinical consequences of inadequate cystine depletion therapy.

Study	Kidney Outcome	Extra-Renal Outcome	Reference
US study; children treated with cysteamine up to 73 months (*n* = 93)	Mean creatinine clearance reduced in patients not adequately depleted (< 1 vs. > 3 nmol half-cystine/mg protein: 50.5 vs. 29.7 mL per min per 1.73 m^2^)		[53]
US single center study; patients analyzed between 1960 and 1992 (*n* = 76)	Non-adequate treatment as shown by leucocyte cystine levels > 2 nmol half-cystine/mg protein. Treatment started at > 2 y with reduced creatinine clearance and early onset of renal impairment (mean creatinine clearance predicted = 0 ml at the age of 20 vs. 74 y for children receiving adequate treatment compared to those with only partial treatment)		[52]
US database; age 18–45 y analyzed between January 1985 and May 2006 (*n* = 100)	Non-adequate treatment, highlighted by leucocyte cystine levels above the cut-off, need for kidney transplantation at least 3.8 y earlier	Non-adequate treatment associated with an increased incidence of hypothyroidism (87% vs. 56%) and death (49% vs. 8%)	[16]
French study; age ≥ 15 y, diagnosis time: 1961–1995, mean follow-up 24.6 y (*n* = 86)	Patients with leucocyte cystine levels > 3 nmol half-cystine/mg protein show early onset ESRD compared to adequately depleted patients (*p* < 0.0001)		[14]
Turkish study; single center retrospective study, age 0.5–29 y, median follow-up 8 y (*n* = 21)		Non-adequate treatment associated with an increased incidence of complications; < 2 vs. ≥2 nmol half-cystine/mg protein with deficit of growth (66.6% vs. 90.9%), pubertal delay (0% vs 66.6%), hypothyroidism (33.3% vs. 54.5%), and diabetes (0% vs 18.1%)	[6]
US database; age 11–48 y analyzed between 1975 and 2005 (*n* = 147)	Adequate treatment associated with delayed onset ESRD (R^2^ = 0.997) Every year of sub-optimal treatment corresponded to a loss of 0.9 y of renal glomerular function 21 patients (born in the 1960s and 1970s) reached ESKD in the first 8 years of life	Mean leucocyte cystine levels (*p* = 0.01), and earlier initiation of cysteamine therapy (*p* = 0.03) significantly associated with improved growth Children reaching CKD stage 5 before 15 years of age grew on average 0.55 height standard deviation scores worse than children that reached dialysis after their 15th birthday (95% CI: 0.09–1.01; *p* = 0.03)	[45]
International European cohort of patients born between 1964 and 2016 (*n* = 453)	Earlier age at start of cysteamine and lower mean leucocyte cystine levels are associated with delayed development of CKD stage 5		[37]

**Table 2:** Novel biomarkers for determination of cystine content in cells and tissues.

Biomarker	Cell/Tissue	Reference
Chitotriosidase	Macrophages	[58]
Alpha-ketoglutarate	Plasma	[67]
Cystine crystals	Skin	[55,69,70]

## Data Availability

Not applicable.

## References

[1] Thoene J., Lemons R., Anikster Y., Mullet J., Paelicke K., Lucero C., Gahl W., Schneider J., Shu S.G., Campbell H.T. (1999). Mutations of CTNS causing intermediate cystinosis. Mol. Genet. Metab..

[2] Town M., Jean G., Cherqui S., Attard M., Forestier L., Whitmore S.A., Callen D.F., Gribouval O., Broyer M., Bates G.P. (1998). A novel gene encoding an integral membrane protein is mutated in nephropathic cystinosis. Nat. Genet..

[3] The Cystinosis Collaborative Research Group (1995). Linkage of the gene for cystinosis to markers on the short arm of chromosome 17. Nat. Genet..

[4] Veys K.R., Elmonem M.A., Arcolino F.O., van den Heuvel L., Levtchenko E. (2017). Nephropathic cystinosis: An update. Curr. Opin. Pediatr..

[5] Besouw M.T., Van Dyck M., Cassiman D., Claes K.J., Levtchenko E.N. (2015). Management dilemmas in pediatric nephrology: Cystinosis. Pediatr. Nephrol..

[6] Gultekingil Keser A., Topaloglu R., Bilginer Y., Besbas N. (2014). Long-term endocrinologic complications of cystinosis. Minerva. Pediatr..

[7] Emma F., Nesterova G., Langman C., Labbe A., Cherqui S., Goodyer P., Janssen M.C., Greco M., Topaloglu R., Elenberg E. (2014). Nephropathic cystinosis: An international consensus document. Nephrol. Dial. Transpl..

[8] Gahl W.A., Thoene J.G., Schneider J.A. (2002). Cystinosis. N. Engl. J. Med..

[9] Topaloglu R. (2021). Nephropathic cystinosis: An update on genetic conditioning. Pediatr. Nephrol..

[10] David D., Princiero Berlingerio S., Elmonem M.A., Oliveira Arcolino F., Soliman N., van den Heuvel B., Gijsbers R., Levtchenko E. (2019). Molecular Basis of Cystinosis: Geographic Distribution, Functional Consequences of Mutations in the CTNS Gene, and Potential for Repair. Nephron..

[11] Ivanova E.A., Arcolino F.O., Elmonem M.A., Rastaldi M.P., Giardino L., Cornelissen E.M., van den Heuvel L.P., Levtchenko E.N. (2016). Cystinosin deficiency causes podocyte damage and loss associated with increased cell motility. Kidney Int..

[12] Levtchenko E., Monnens L. (2006). Development of Fanconi syndrome during infancy in a patient with cystinosis. Acta. Paediatr..

[13] Long W.S., Seashore M.R., Siegel N.J., Bia M.J. (1990). Idiopathic Fanconi syndrome with progressive renal failure: A case report and discussion. Yale. J. Biol. Med..

[14] Brodin-Sartorius A., Tete M.J., Niaudet P., Antignac C., Guest G., Ottolenghi C., Charbit M., Moyse D., Legendre C., Lesavre P. (2012). Cysteamine therapy delays the progression of nephropathic cystinosis in late adolescents and adults. Kidney Int..

[15] Nesterova G., Gahl W.A. (2013). Cystinosis: Tthe evolution of a treatable disease. Pediatr. Nephrol..

[16] Gahl W.A., Balog J.Z., Kleta R. (2007). Nephropathic cystinosis in adults: Natural history and effects of oral cysteamine therapy. Ann. Intern. Med..

[17] Schiefer J., Zenker M., Grone H.J., Chatzikyrkou C., Mertens P.R., Liakopoulos V. (2018). Unrecognized juvenile nephropathic cystinosis. Kidney. Int..

[18] Manz F., Gretz N. (1985). Cystinosis in the Federal Republic of Germany. Coordination and analysis of the data. J. Inherit. Metab. Dis..

[19] Cherqui S., Courtoy P.J. (2017). The renal Fanconi syndrome in cystinosis: Pathogenic insights and therapeutic perspectives. Nat. Rev. Nephrol..

[20] Greco M., Brugnara M., Zaffanello M., Taranta A., Pastore A., Emma F. (2010). Long-term outcome of nephropathic cystinosis: A 20-year single-center experience. Pediatr. Nephrol..

[21] Schulman J.D., Wong V.G., Kuwabara T., Bradley K.H., Seegmiller J.E. (1970). Intracellular cystine content of leukocyte populations in cystinosis. Arch. Intern. Med..

[22] Schneider J.A., Bradley K., Seegmiller J.E. (1967). Increased cystine in leukocytes from individuals homozygous and heterozygous for cystinosis. Science.

[23] Oshima R.G., Willis R.C., Furlong C.E., Schneider J.A. (1974). Binding assays for amino acids. The utilization of a cystine binding protein from Escherichia coli for the determination of acid-soluble cystine in small physiological samples. J. Biol. Chem..

[24] Smith M., Furlong C.E., Greene A.A., Schneider J.A. (1987). Cystine: Binding protein assay. Methods Enzymol..

[25] Chabli A., Aupetit J., Raehm M., Ricquier D., Chadefaux-Vekemans B. (2007). Measurement of cystine in granulocytes using liquid chromatography-tandem mass spectrometry. Clin. Biochem..

[26] de Graaf-Hess A., Trijbels F., Blom H. (1999). New method for determining cystine in leukocytes and fibroblasts. Clin. Chem..

[27] Elmonem M.A., Veys K.R., Soliman N.A., van Dyck M., van den Heuvel L.P., Levtchenko E. (2016). Cystinosis: A review. Orphanet. J. Rare. Dis..

[28] Smolin L.A., Clark K.F., Schneider J.A. (1987). An improved method for heterozygote detection of cystinosis, using polymorphonuclear leukocytes. Am. J. Hum. Genet..

[29] Levtchenko E., de Graaf-Hess A., Wilmer M., van den Heuvel L., Monnens L., Blom H. (2004). Comparison of cystine determination in mixed leukocytes vs polymorphonuclear leukocytes for diagnosis of cystinosis and monitoring of cysteamine therapy. Clin. Chem..

[30] Fowler B., Bielsky M.C., Farrington Z. (2001). White Cell Cystine Group.

[31] Chadefaux-Vekemans B. (2001). White Cell Cystine Group: Guideline no. 2. Polymorphonuclear Leucocyte Preparation.

[32] Gertsman I., Johnson W.S., Nishikawa C., Gangoiti J.A., Holmes B., Barshop B.A. (2016). Diagnosis and Monitoring of Cystinosis Using Immunomagnetically Purified Granulocytes. Clin. Chem..

[33] Lowry O.H., Rosebrough N.J., Farr A.L., Randall R.J. (1951). Protein measurement with the Folin phenol reagent. J. Biol. Chem..

[34] Smith P.K., Krohn R.I., Hermanson G.T., Mallia A.K., Gartner F.H., Provenzano M.D., Fujimoto E.K., Goeke N.M., Olson B.J., Klenk D.C. (1985). Measurement of protein using bicinchoninic acid. Anal. Biochem..

[35] Powell K.L., Langman C.B. (2012). An unexpected problem in the clinical assessment of cystinosis. Pediatr. Nephrol..

[36] Langman C.B., Barshop B.A., Deschenes G., Emma F., Goodyer P., Lipkin G., Midgley J.P., Ottolenghi C., Servais A., Soliman N.A. (2016). Controversies and research agenda in nephropathic cystinosis: Conclusions from a “Kidney Disease: Improving Global Outcomes” (KDIGO) Controversies Conference. Kidney. Int..

[37] Emma F., Hoff W.V., Hohenfellner K., Topaloglu R., Greco M., Ariceta G., Bettini C., Bockenhauer D., Veys K., Pape L. (2021). An international cohort study spanning five decades assessed outcomes of nephropathic cystinosis. Kidney. Int..

[38] Jackson M., Young E. (2005). Prenatal diagnosis of cystinosis by quantitative measurement of cystine in chorionic villi and cultured cells. Prenat. Diagn..

[39] da Silva V.A., Zurbrugg R.P., Lavanchy P., Blumberg A., Suter H., Wyss S.R., Luthy C.M., Oetliker O.H. (1985). Long-term treatment of infantile nephropathic cystinosis with cysteamine. N. Engl. J. Med..

[40] Gahl W.A., Thoene J.G., Scriver C.R., Sly W.S., Childs B., Beaudet A.L., Valle D., Kinzler K.W., Vogelstein B. (2001). Cystinosis: A disorder of lysosomal membrane transport. The Metabolic and Molecular Bases of Inherited Disease.

[41] Surmeli Doven S., Delibas A., Kayacan U.R., Unal S. (2017). Short-cut diagnostic tool in cystinosis: Bone marrow aspiration. Pediatr. Int..

[42] Wamelink M.M., Struys E.A., Jansen E.E., Blom H.J., Vilboux T., Gahl W.A., Komhoff M., Jakobs C., Levtchenko E.N. (2011). Elevated concentrations of sedoheptulose in bloodspots of patients with cystinosis caused by the 57-kb deletion: Implications for diagnostics and neonatal screening. Mol. Genet. Metab..

[43] Fleige T., Burggraf S., Czibere L., Haring J., Gluck B., Keitel L.M., Landt O., Harms E., Hohenfellner K., Durner J. (2020). Next generation sequencing as second-tier test in high-throughput newborn screening for nephropathic cystinosis. Eur. J. Hum. Genet..

[44] Hohenfellner K., Bergmann C., Fleige T., Janzen N., Burggraf S., Olgemoller B., Gahl W.A., Czibere L., Froschauer S., Roschinger W. (2019). Molecular based newborn screening in Germany: Follow-up for cystinosis. Mol. Genet. Metab. Rep..

[45] Nesterova G., Williams C., Bernardini I., Gahl W.A. (2015). Cystinosis: Renal glomerular and renal tubular function in relation to compliance with cystine-depleting therapy. Pediatr. Nephrol..

[46] Servais A., Saitovitch A., Hummel A., Boisgontier J., Scemla A., Sberro-Soussan R., Snanoudj R., Lemaitre H., Legendre C., Pontoizeau C. (2020). Central nervous system complications in adult cystinosis patients. J. Inherit. Metab. Dis..

[47] Wilmer M.J., Schoeber J.P., van den Heuvel L.P., Levtchenko E.N. (2011). Cystinosis: Practical tools for diagnosis and treatment. Pediatr. Nephrol..

[48] Levtchenko E.N., van Dael C.M., de Graaf-Hess A.C., Wilmer M.J., van den Heuvel L.P., Monnens L.A., Blom H.J. (2006). Strict cysteamine dose regimen is required to prevent nocturnal cystine accumulation in cystinosis. Pediatr. Nephrol..

[49] Linden S., Klank S., Harms E., Gruneberg M., Park J.H., Marquardt T. (2020). Cystinosis: Therapy adherence and metabolic monitoring in patients treated with immediate-release cysteamine. Mol. Genet. Metab. Rep..

[50] Ariceta G., Giordano V., Santos F. (2019). Effects of long-term cysteamine treatment in patients with cystinosis. Pediatr. Nephrol..

[51] Kleta R., Kaskel F., Dohil R., Goodyer P., Guay-Woodford L.M., Harms E., Ingelfinger J.R., Koch V.H., Langman C.B., Leonard M.B. (2005). Diseases, N.I.H.O.o.R. First NIH/Office of Rare Diseases Conference on Cystinosis: Past, present, and future. Pediatr. Nephrol..

[52] Markello T.C., Bernardini I.M., Gahl W.A. (1993). Improved renal function in children with cystinosis treated with cysteamine. N. Engl. J. Med..

[53] Gahl W.A., Reed G.F., Thoene J.G., Schulman J.D., Rizzo W.B., Jonas A.J., Denman D.W., Schlesselman J.J., Corden B.J., Schneider J.A. (1987). Cysteamine therapy for children with nephropathic cystinosis. N. Engl. J. Med..

[54] Labbe A., Niaudet P., Loirat C., Charbit M., Guest G., Baudouin C. (2009). In vivo confocal microscopy and anterior segment optical coherence tomography analysis of the cornea in nephropathic cystinosis. Ophthalmology.

[55] Chiaverini C., Kang H.Y., Sillard L., Berard E., Niaudet P., Guest G., Cailliez M., Bahadoran P., Lacour J.P., Ballotti R. (2013). In vivo reflectance confocal microscopy of the skin: A noninvasive means of assessing body cystine accumulation in infantile cystinosis. J. Am. Acad. Dermatol..

[56] Elmonem M.A., Makar S.H., van den Heuvel L., Abdelaziz H., Abdelrahman S.M., Bossuyt X., Janssen M.C., Cornelissen E.A., Lefeber D.J., Joosten L.A. (2014). Clinical utility of chitotriosidase enzyme activity in nephropathic cystinosis. Orphanet. J. Rare. Dis..

[57] Prencipe G., Caiello I., Cherqui S., Whisenant T., Petrini S., Emma F., De Benedetti F. (2014). Inflammasome activation by cystine crystals: Implications for the pathogenesis of cystinosis. J. Am. Soc. Nephrol..

[58] Veys K.R.P., Elmonem M.A., Van Dyck M., Janssen M.C., Cornelissen E.A.M., Hohenfellner K., Prencipe G., van den Heuvel L.P., Levtchenko E. (2020). Chitotriosidase as a Novel Biomarker for Therapeutic Monitoring of Nephropathic Cystinosis. J. Am. Soc. Nephrol..

[59] Lobry T., Miller R., Nevo N., Rocca C.J., Zhang J., Catz S.D., Moore F., Thomas L., Pouly D., Bailleux A. (2019). Interaction between galectin-3 and cystinosin uncovers a pathogenic role of inflammation in kidney involvement of cystinosis. Kidney Int..

[60] Brown R.J. (1952). A clinico-pathological study of cystinosis in two siblings. Arch. Dis. Child..

[61] DiDomenico P., Berry G., Bass D., Fridge J., Sarwal M. (2004). Noncirrhotic portal hypertension in association with juvenile nephropathic cystinosis: Case presentation and review of the literature. J. Inherit. Metab. Dis..

[62] Dohil R., Carrigg A., Newbury R. (2012). A potential new method to estimate tissue cystine content in nephropathic cystinosis. J. Pediatr..

[63] Guillet G., Sassolas B., Fromentoux S., Gobin E., Leroy J.P. (1998). Skin storage of cystine and premature skin ageing in cystinosis. Lancet.

[64] Monier L., Mauvieux L. (2015). Cystine crystals in bone marrow aspirate. Blood.

[65] Elmonem M.A., Ramadan D.I., Issac M.S., Selim L.A., Elkateb S.M. (2014). Blood spot versus plasma chitotriosidase: A systematic clinical comparison. Clin. Biochem..

[66] Guo Y., He W., Boer A.M., Wevers R.A., de Bruijn A.M., Groener J.E., Hollak C.E., Aerts J.M., Galjaard H., van Diggelen O.P. (1995). Elevated plasma chitotriosidase activity in various lysosomal storage disorders. J. Inherit. Metab. Dis..

[67] Jamalpoor A., van Gelder C.A., Yousef Yengej F.A., Zaal E.A., Berlingerio S.P., Veys K.R., Pou Casellas C., Voskuil K., Essa K., Ammerlaan C.M. (2021). Cysteamine-bicalutamide combination therapy corrects proximal tubule phenotype in cystinosis. EMBO Mol. Med..

[68] Elmonem M.A., Veys K., Oliveira Arcolino F., Van Dyck M., Benedetti M.C., Diomedi-Camassei F., De Hertogh G., van den Heuvel L.P., Renard M., Levtchenko E. (2018). Allogeneic HSCT transfers wild-type cystinosin to nonhematological epithelial cells in cystinosis: First human report. Am. J. Transplant..

[69] Veys K.R.P., Elmonem M.A., Dhaenens F., Van Dyck M., Janssen M., Cornelissen E.A.M., Hohenfellner K., Reda A., Quatresooz P., van den Heuvel B. (2019). Enhanced Intrinsic Skin Aging in Nephropathic Cystinosis Assessed by High-Definition Optical Coherence Tomography. J. Investig. Dermatol..

[70] Bengali M., Goodman S., Sun X., Dohil M.A., Dohil R., Newbury R., Lobry T., Hernandez L., Antignac C., Jain S. (2021). Non-invasive intradermal imaging of cystine crystals in cystinosis. PLoS ONE.

